# Do Parental Perceptions of the Nutritional Quality of School Meals Reflect the Food Environment in Public Schools?

**DOI:** 10.3390/ijerph182010764

**Published:** 2021-10-14

**Authors:** Sarah Martinelli, Francesco Acciai, Michael J. Yedidia, Punam Ohri-Vachaspati

**Affiliations:** 1 College of Health Solutions, Arizona State University, Phoenix, AZ 85004, USA; Facciai@asu.edu (F.A.); Punam.Ohri-Vachaspati@asu.edu (P.O.-V.); 2 Rutgers Center for State Health Policy, Institute for Health, Rutgers University, New Brunswick, NJ 08901, USA; myedidia@ifh.rutgers.edu

**Keywords:** school food, HHFKA, nutrition, perceptions

## Abstract

(1) Background: It is unknown whether parents’ perception of school meals, a determinant of student meal participation, align with the nutritional quality of meals served in schools. This study compares the healthfulness of foods offered in schools with parental perception of school meals at those same schools. (2) Method: Parents were asked to rate the healthfulness of school meals at their child’s school. Data on the types of foods offered were collected from public schools in four cities in New Jersey and matched with parent-reported data. Measures were developed to capture the presence of healthy and unhealthy items in the National School Lunch Program and the presence of a la carte offerings as well as vending machines. Multivariable analysis examined the association between parental perceptions of school meals and the school food measures after adjusting for covariates. (3) Results: Measures of the school food environment and parental perceptions were available for 890 pre-K to 12th grade students. No significant associations were observed between parental perceptions and food environment measures when examined one by one or in a comprehensive model. (4) Conclusions: Parents’ perception of the healthfulness of meals served do not align with the nutritional quality of foods offered at schools.

## 1. Introduction

The Healthy Hunger Free Kids Act (HHFKA), the first major update to the National School Lunch Program (NSLP) since the 1990s, was approved by congress in 2010 and implemented in a stepwise fashion in schools starting in the 2012–2013 school year (SY) [1]. The HHFKA focused on improving the nutritional quality of the foods provided to children through the NSLP by setting age-specific calorie, sodium, and fat maximums as well as requiring more whole grains and a greater variety of fresh fruits and vegetables to be served each week [1]. Similar regulations were applied to competitive foods (i.e., foods sold in schools outside of the NSLP, such as a la carte and in vending machines) starting in SY 2014–2015, with the Smart Snacks Standards [2]. Since its implementation, the positive impact of the HHFKA on the nutritional quality of the foods served and sold in schools has been well documented [3,4,5,6]. Studies have consistently shown that children who eat school lunches are selecting and consuming more nutrient-dense meals [4], including more fruits, vegetables and whole grains [6,7,8], while school meal participation rates have remained steady [9], indicating students’ acceptance of these meals. Children who eat school meals also tend to have healthier overall diets compared to their non-participating counterparts [10,11,12], and meals served in schools are healthier than those brought from home [13,14,15]. Further, a recent study examining trends in foods consumed by children ages 5–19 found that the nutrition quality of foods consumed at schools significantly improved after 2010 and that these foods had higher nutrition quality compared to foods consumed from other locations such as grocery stores and restaurants [6].

Despite marked improvements in the nutritional quality of school meals, school meal participation did not increase; both prior to and after implementation of the HHFKA, participation rates were, and remained around 70% [4,9]. Similarly, parental perceptions of the healthfulness of school meals—a predictor of students’ school meal participation—did not change after implementation of the HHFKA [16], with parental assessment of school meals remaining low [17,18]. Even though parents’ impressions of the nutritional quality of school meals plays a critical role in whether or not a child participates in the program [16,19], no studies to date have examined how parental assessment of the nutritional quality of school meals compares with the healthfulness of the food actually served at the school their child attends. To address this gap, the goal of the current study was to examine the association between parent perceptions of the healthfulness of school meals and the food offerings at their child’s school. This is critical for designing interventions aimed at increasing participation in school meal programs, which are a major source of healthy nutrition for school-aged children. We hypothesize that there is an overall misalignment of parental perceptions of school meal healthfulness with measures of the nutritional quality of school meals in the school their child attends. However, based on indications that parents of younger students are more engaged with their children’s school activities, we expect a more accurate alignment between perceptions and the school food environment among parents of elementary school children.

## 2. Materials and Methods

This is a secondary analysis using data from the ongoing New Jersey Child Health Study (NJCHS). The NJCHS investigates the impact of the food and physical activity environment on children’s weight and health outcomes over time. The study collected data from a sample of households with children located in four predominately low-income cities in New Jersey: Camden, New Brunswick, Newark, and Trenton. Data were also collected on the school food environment in all public schools in the four study cities through a school survey. Both household and school survey respondents were compensated for their time. The study was approved by the Institutional Review Boards of Rutgers and Arizona State Universities.

### 2.1. Household Survey

Computer-assisted phone interviews were used to collect data from two panels of households with children at two times points between 2009 and 2017. Data collected at time 1 on each panel (2009–2010 and 2014–2015) were included in the current analysis. In panel one, households were selected using a random digit dialing of landline telephone numbers associated with the study cities. In panel two, cell phones were added to the sampling design to reflect the increased use of cell phones over landlines. Households were eligible if they lived within the study city limits, had a child in the home between the ages of 3 and 18, and spoke English or Spanish. The respondent was an adult, at least 18 years old, who provided responses for themselves and one randomly selected child (referred to as the index child) in the household. Additional details about the household survey design are available elsewhere [20].

### 2.2. Household Survey Content

Parents reported socio-economic and demographic characteristics of the household, including household income and mother’s education, as well as age, sex, and ethnicity/race of the index child. Respondents also provided the name of the school the child attended at the time of the survey. Children were grouped into 3 race/ethnicity categories—Hispanic, non-Hispanic Black, and non-Hispanic White/other. Mother’s education was categorized as less than high school, high school, and at least some college. To measure participation in school meals, parents were asked, “On most days, does (index child) have a lunch served by the school?” with yes and no as response options [21]. To assess parents’ perception of the school meals, parents were asked: “Regardless of whether or not (index child) eats foods provided by his/her school, how would you rate the nutritional quality of foods offered at (index child’s) school?” Answer options were on a 4-point Likert scale: “Very Unhealthy”, “Unhealthy”, “Healthy” and “Very Healthy” with the option to refuse or select “I don’t know” or “School does not provide food.”

### 2.3. School-Level Data

A survey using questions from prior research was used to gather information on specific aspects of the food environments in schools [22,23,24]. The survey included questions about foods offered as part of reimbursable school lunches, a la carte during lunch time, and in vending machines. Surveys developed using Qualtrics^®^ (Provo, UT, USA) were distributed in paper and online formats to school nurses in all public schools in our study cities that included any grade from K to 12. School nurses were asked to draw upon their own knowledge as well as consult with school food staff to complete the survey. Data about school environments were collected for each SY between 2010–2011 and 2017–2018. The current analysis used 2010–2011 and 2014–2015 school environment data for the first and second panels of the household survey, respectively. Overall, the response rate to the school environment survey averaged 92.5% across all schools in the four districts. We were able to match data for 96 schools for SY 2010–2011 and for 88 schools for SY 2014–2015 to the household data.

Data from the school food environment survey were summarized in a series of indices. For this analysis we used four indices: NSLP healthy; NLSP unhealthy; presence of vending machines; presence of a la carte items served in the cafeteria during lunch. The NSLP healthy index (range 0–9) indicates the total number of healthy items (e.g., whole grains, salad bar, fresh fruits, etc.) offered as part of the NLSP. The NSLP unhealthy food index consisted of similar counts of available food items that were designated as unhealthy (e.g., fries, dessert, pizza, etc.) and could range from 0 to 5. A complete list of the items included in these two indices is provided in Table 1. Taken together, they represent the overall exposure to healthy and unhealthy items offered in school meals. To capture competitive foods, two binary variables were created to indicate (1) whether there were vending machines available to children in school, and (2) whether a la carte items were served during school meals. While we collected data on the number of healthy and unhealthy items offered in these two venues, use of the binary variables was preferred for two reasons: first, there was a relatively large number of schools that did not have vending machines (54%), or a la carte (29%), thus scoring 0 on both healthy and unhealthy items. Second, schools that had vending machines and/or a la carte offerings, tended to have a similar proportion of healthy and unhealthy items, resulting in high correlations between the numbers of healthy and unhealthy items offered through each of these venues. More detail about the school environment survey and index development can be found elsewhere [25].

In addition to school data collected via the school environment survey, school characteristics such as total student enrollment, racial/ethnic composition, and proportion of children eligible for free or reduced-price meals (FRPM) were taken from the National Center for Education Statistics [26] and were included in the analysis as control variables.

### 2.4. Analytical Sample

The analytical sample consisted of 1,201 students (from both time points) who attended public schools in the study cities. A total of 311 responses were excluded due to missing data on the school food environment (n = 184), parent perceptions (n = 108), or other variables (n = 19). The final analytical sample included 890 respondents with complete data on variables included in the analysis. The cases that were excluded from the final analytical sample did not differ from those in the analytical sample on any individual or household characteristics, except for race. The cases that were excluded consisted of a higher proportion of non-Hispanic Black children and a lower proportion of Hispanic and non-Hispanic White/other children.

### 2.5. Data Analysis

Ordered logistic regression was used to examine the association between parental perception of the healthfulness of school meals and each of the school food environment indices individually. Next, all four indices were entered in the model together to assess a comprehensive account of the school food environment. Each model controlled for age, sex, and race of the child, household income as a ratio to the federal poverty line, and mother’s education. Models also adjusted for school-level factors (i.e., school size, racial composition, proportion of students eligible for FRPM, and whether the school was an elementary school or a middle/high school). An indicator for panel was also used as a control variable to account for any potential unaccounted differences across the two panels, given that panel one was collected before HHFKA and panel two after HHFKA implementation. Lastly, all models were adjusted for clustering at the city level and included sampling weights to account for the complex survey design and to ensure the sample was representative of the cities from which it was taken. To test if the relationship between the school food environment (captured by the four indices) and parents’ perception of the healthfulness of school meals was moderated by school level (elementary vs. middle/high school), interaction terms representing school level and each of the four indices (one at a time) were added to the comprehensive model. In additional models, similar analyses were conducted by introducing interaction terms between panel and each of the four indices to assess whether the relationship between parental perception of the healthfulness of the school food environment and measures of that environment changed between the two panels. Sensitivity analyses were run after recoding the perception variable into two and three categories. Additional sensitivity analyses were conducted to examine differences in associations by race/ethnicity and student school meal participation status in regression models. All analyses were run using Stata 15.1 (StataCorp LLC, College Station, TX, USA, 2017).

## 3. Results

### 3.1. Descriptive Statistics

Table 2 shows the demographic information for the sample including child, household, and school level variables. Most parents (72%) perceived school meals to be either “somewhat healthy” or “very healthy” while 28% of parents perceived school meals to be either “somewhat unhealthy” or “very unhealthy.” The sample was comprised primarily of non-Hispanic Black and Hispanic children (50% and 44%, respectively). The average age of the children was 10.8 years, and the majority of them (66%) attended an elementary school. Nearly all children (91%) participated in school meals.

### 3.2. Relationship between School Food Environment and Perceptions

As shown in Table 3, in multivariable models, none of the school food environment indices were significantly associated with parental perceptions of school meals when examined individually (models 1–4). Only for NSLP healthy did the association approach significance; for every additional healthy item served in the NSLP, parents were 14% (*p* = 0.054) more likely to have a more positive perception of the school meals (OR 1.14; CI: 1.00–1.29). The comprehensive model that examined all 4 indices of the school food environment together (model 5) showed results that were consistent with models 1–4. Across all models, parents of non-Hispanic Black children and children who participated in school meals were significantly more likely to give a more positive assessment of the healthfulness of the school food environment.

### 3.3. Subgroup Analyses 

The association between parents’ perception and the school food environment indices did not significantly vary by school level (Table 4). However, presence of vending machines was marginally associated with more negative perceptions of the food environment for parents of elementary school children. The results were similar to those presented in Table 3 when interaction terms between panel and indices were introduced in the models. The association between parent perception and school food environment indices were not significant either before or after implementation of the HHFKA.

Sensitivity analyses using the perception variable based on two categories (combining “very unhealthy” with “unhealthy” and “healthy” with “very healthy”) or three categories (combining “very unhealthy” with “unhealthy” and leaving “healthy” and “very healthy” separate), using a logit and an ordinal logit model, respectively, yielded similar results. Additional sensitivity analyses examining differences by race and student participation in school meals also resulted in similar findings; with the lack of association between parental perceptions and food indices observed across all racial/ethnic groups and independent of children school meal participation status. 

## 4. Discussion

This study examined the relationship between parents’ perception of school meals and the healthfulness of the school food environment in public schools in four cities with low-income and high minority populations in New Jersey. Consistent with our hypothesis, parental perceptions of school meals were not associated with actual measurements of the school food environment. None of the four measures of the food environment were associated with parents’ perception when examined alone or together in a comprehensive model. Results were similar across school levels and time periods—before and after implementation of the HHFKA. Parents of non-Hispanic Black children and of those who participated in school meals tended to have an overall more positive perception of school meals; nevertheless, interaction analyses showed that their perceptions of the healthfulness of the school meals were not aligned with the nutritional quality of the meals served. The current findings underscore the disconnect between school meals and parents’ perception of those meals, highlighting the importance of efforts to better acquaint parents with the nutritional quality of school meals. Our findings might help explain why prior research has shown that parents’ perception of school meals did not improve after the implementation of the HHFKA [16], despite improvements in the overall nutritional quality of school meals [3,4,5,6].

The presence of competitive foods, specifically those served a la carte and in vending machines, was also not associated with parental perceptions of school meals. Competitive foods typically include snack foods such as chips, cookies and ice cream—all items for which there were no nutritional standards until implementation of the Smart Snacks standards in 2014 [2]. After 2014, schools were required to offer competitive foods that met nutrition standards similar to those of the NSLP, including whole grain requirements and setting limits on calories, sodium, and fat [2]. How schools responded to these requirements may have contributed to parents’ misperception of the healthfulness of competitive foods. For example, school specific versions of popular snacks were created by food manufacturers, often referred to as “look alike” products [27]. As a result, chips sold in schools might have less fat and salt than the non-school version of the same item sold at a local grocery store. While the use of “look alike” products does result in an improvement of the nutritional quality of competitive foods in both venues (vending machines and a la carte), that improvement may not be immediately obvious to students or parents [27]. The use of “look alike” products is not limited to competitive foods. For instance, some popular items served as part of the NSLP, including chicken nuggets and pizza, fall within this category. While these items do meet healthier nutrition guidelines post HHFKA, it may be difficult for parents to recognize their nutritional benefits. It is also possible that the lack of association between the presence of competitive foods (especially vending) and parents’ perception is impacted by the relatively low prevalence of vending machines in our sample (54% of schools did not have vending machines).

Prior research shows that participation in school meals tends to be higher for younger children compared to older children [5,28], and parents of younger children are likely to be more engaged with school activities [29] and more familiar with the school environment compared to parents of older children. It was therefore hypothesized that there would be better alignment between perceptions and actual school food environment among parents of elementary school children as compared to parents of middle/high school students. Contrary to expectations, the results from this study did not suggest any moderation effect of school level on the association between perceptions and the school food environment.

These findings highlight the misalignment between the healthfulness of the school food environment and parents’ views of food offered in school. Prior research has shown that parents’ perception of school meals did not improve after the implementation of the HHFKA [16] despite improvements in the healthfulness of school meals [25]. This is a troubling issue, as participation in school meals is impacted by parental perceptions of those meals [16,19] and could, at least partially, account for the fact that on an average school day, only 56% of students participated in the NLSP [5]. Based on the most recently available data, not all students who are eligible for FRPM participate in meals on a regular basis [28]. For instance, in the 2009–2010 school year, 79.1% of students eligible for free meals and 73.2% of students eligible for reduced-price meals participated in the NSLP [28]. The goal of the NSLP to reduce food and nutrition insecurity by providing healthy meals to low-income children can only be achieved if children participate in school meals.

In addition to missed opportunities for improving children’s nutrition, sub-optimal participation in school meal programs may impede schools’ abilities to meet the fixed costs of maintaining a viable meals service (e.g., costs of equipment and personnel). The main source of funding for school food programs is through federal reimbursement for meals served. These funds are used not only to purchase food but also for equipment and wages for employees who prepare and serve meals. The latter expenses often do not vary with the volume of meals served; yet, low participation numbers affect a program’s ability to meet these costs and successfully provide meals to students. In addition to the impact on participation, lack of understanding about the nutritional quality of school meals by parents and other stakeholders, including teachers and administrators, may result in less support from these groups for school food programs. The current findings suggest the need for effective efforts to acquaint parents with the quality of food served at their children’s schools through frequent and effective communication using multiple channels. These might include family meal days when parents join students for lunch, inclusion of more nutrition information in menus distributed to parents, newsletters featuring school foods, use of social media to discuss school meals, and other targeted marketing strategies.

### Limitations

Among the study’s limitations, a bias toward endorsing socially desirable responses may have impacted parents’ reported perception of the nutritional quality of school meals, given the high participation rate in school meals in our sample. To account for this potential bias, the analysis adjusted for school meal participation by the index child. Our analysis is based on a cross-sectional design; therefore, the associations observed cannot be considered causal. We do not have information on parents’ nutrition knowledge, which may influence their understanding and perception of nutritional quality of the school meals. In addition, our findings may not be generalizable beyond communities similar to our study cities—i.e., low-income, racially/ethnically diverse populations. Further, as this is a secondary data analysis, we were not able to collect qualitative data, which may have provided more details about the origins of parents’ thoughts and beliefs about school meals. Lastly, we were not able to gather detailed information from schools about their use of health education classes or other health related information aimed at parents.

## 5. Conclusions

Parents’ perception of school meals are not aligned with measures of the healthfulness of school food offerings. This disconnect can impact participation rates in school meals and undermine the potential to reduce food insecurity and improve children’s diet quality by providing nutritious meals to students. School-, state-, and federal-level stakeholders could improve communication with parents about the nutritional content of school meals to address this misperception. School food departments could also consider including students and parents in recipe and product testing so their opinions and views are better incorporated into menu decisions. Focused marketing strategies highlighting the healthfulness of the resulting menus could be incorporated as well.

## Figures and Tables

**Table 1:** List of items included in indices capturing the food environment in the National School Lunch Program (NSLP) in K-12 schools.

Index Name and Score Range	Items Included
National School Lunch Program (NSLP) Healthy (0–9)	At least half whole grains
	Whole grains
	Variety of vegetables
	Modified pizza
	Fat free/1% milk
	Fat free flavored milk
	Fresh fruit
	Raw vegetables
	Salad bar
National School Lunch Program (NSLP) Unhealthy (0–5)	Fries
	Pizza
	Dessert
	Full fat/2% milk
	Full fat/1% flavored milk

**Table 2:** Descriptive characteristics of the analytical sample (N = 890).

		% or Mean (SD)
Perception of healthfulness of school meals	Very unhealthy	10.1
	Somewhat unhealthy	18.1
	Somewhat healthy	49.2
	Very healthy	22.6
**Child Level Factors**		
Ethnicity/race	Non-Hispanic White/other	6.3
	Non-Hispanic Black	49.6
	Hispanic	44.2
Sex	Male	50.8
	Female	49.2
Age (years)		10.8(3.7)
Participation in school meals	No	9.6
	Yes	90.5
School level attended	Elementary	65.8
	Middle/high school	34.2
**Household Level Factors**		
Mother’s education	Less than HS	24.9
	HS or equivalent	42.1
	At least some college	32.9
Poverty level (% of FPL)		189(4.0)
**School Level Factors**		
Total enrollment		643(338)
Free and reduced-price meal eligibility		81.7(12.9)

**Table 3:** Results from multivariable ordinal logistic regression examining the association between parental perceptions and each school food environment measure alone and all together in the model.

		Model 1			Model 2			Model 3			Model 4			Model 5		
		OR	95% CI		OR	95% CI		OR	95% CI		OR	95% CI		OR	95% CI	
Food Environment Index	NSLP healthy	1.14 ^	1.00	1.29										1.13 ^	0.99	1.29
	NSLP unhealthy				1.05	0.87	1.27							1.06	0.87	1.28
	A la carte presence							1.37	0.88	2.14				1.37	0.86	2.16
	Vending presence										0.78	0.50	1.22	0.72	0.46	1.12
**Child Level Factors**																
Race	Non-Hispanic White/other (Reference)															
	Non-Hispanic Black	2.38 *	1.32	4.27	2.45 *	1.39	4.34	2.32 *	1.30	4.11	2.49 *	1.40	4.43	2.36 *	1.31	4.27
	Hispanic	0.96	0.56	1.67	0.95	0.56	1.62	0.92	0.53	1.59	0.96	0.56	1.64	0.96	0.55	1.68
Sex	Male (Reference)															
	Female	0.75	0.51	1.10	0.74	0.51	1.08	0.74	0.51	1.08	0.74	0.51	1.08	0.75	0.51	1.10
Age		0.97	0.91	1.04	0.97	0.91	1.04	0.97	0.91	1.04	0.98	0.91	1.05	0.98	0.91	1.05
Participation in school meals	No (Reference)															
	Yes	2.94 **	1.66	5.18	2.77 **	1.57	4.90	2.92 **	1.63	5.23	2.76 **	1.58	4.82	2.98 **	1.68	5.28
School level attended	Elementary (Reference)															
	Middle/high school	0.90	0.52	1.58	0.94	0.54	1.64	0.92	0.53	1.59	0.99	0.58	1.70	0.98	0.57	1.68
**Household Level Factors**																
Mother’s education	Less than HS (Reference)															
	HS or equivalent	1.29	0.79	2.09	1.28	0.79	2.07	1.30	0.80	2.10	1.32	0.81	2.13	1.31	0.81	2.11
	At least some college	0.65	0.38	1.13	0.65	0.38	1.11	0.64	0.37	1.10	0.67	0.39	1.13	0.63^	0.37	1.09
Poverty level (% of FPL)		1.02	0.96	1.07	1.01	0.96	1.07	1.02	0.96	1.08	1.01	0.96	1.07	1.02	0.96	1.08
**School Level Factors**																
Total enrollment		1.00	1.00	1.00	1.00	1.00	1.00	1.00	1.00	1.00	1.00	1.00	1.00	1.00	1.00	1.00
Free and reduced-price meal eligibility		3.97	0.65	24.36	3.40	0.58	20.10	3.05	0.52	18.09	3.04	0.51	18.03	2.93	0.47	18.22

^ *p* < 0.10, * = *p* < 0.05, ** = *p* < 0.001; Model 5 is a comprehensive model that includes all four measures of the school food environment.

**Table 4:** Interaction between measures of the school food environment and parental perceptions by school level.

	Elementary			Middle/High			*p* for Interaction
	OR	95% CI		OR	95% CI		
NSLP healthy	1.09	0.93	1.27	1.38 ^	0.97	1.97	0.23
NSLP unhealthy	1.10	0.86	1.41	1.01	0.77	1.34	0.66
A la carte presence	1.30	0.76	2.22	1.25	0.48	3.31	0.95
Vending presence	0.60 ^	0.34	1.08	1.26	0.63	2.52	0.09

The interactions between school level and the four indices of the school food environment were included in the same model. None of the associations were significant at *p* < 0.05; ^ *p* < 0.10.

## Data Availability

The data used in this study is available upon request.
